# Genetic variation of *Mycobacterium tuberculosis *circulating in Kharkiv Oblast, Ukraine

**DOI:** 10.1186/1471-2334-11-77

**Published:** 2011-03-28

**Authors:** Maya A Dymova, Oleksander O Liashenko, Petro I Poteiko, Valeriy S Krutko, Eugeny A Khrapov, Maxim L Filipenko

**Affiliations:** 1 Institute of Chemical Biology and Basic Medicine, Siberian Branch, Russian Academy of Sciences, Novosibirsk, Russian Federation, Russia; 2 Kharkiv Medical Academy of Graduate Education, Kharkiv, Ukraine

## Abstract

**Background:**

A persistent increase of tuberculosis cases has recently been noted in the Ukraine. The reported incidence of drug-resistant isolates of *M. tuberculosis *is growing steadily; however, data on the genetic variation of isolates of *M. tuberculosis *circulating in northern Ukraine and on the spectrum and frequency of occurrence of mutations determining resistance to the principal anti-tuberculosis drugs isoniazid and rifampicin have not yet been reported.

**Methods:**

Isolates of *M. tuberculosis *from 98 tuberculosis patients living in Kharkiv Oblast (Ukraine) were analyzed using VNTR- and RFLP-IS6110-typing methods. Mutations associated with resistance to rifampicin and isoniazid were detected by RFLP-PCR methods, and also confirmed by sequencing.

**Results:**

We identified 75 different genetic profiles. Thirty four (34%) isolates belonged to the Beijing genotype and 23 (23%) isolates belonged to the LAM family. A cluster of isolates belonging to the LAM family had significant genetic heterogeneity, indicating that this family had an ancient distribution and circulation in this geographical region. Moreover, we found a significant percentage of the isolates (36%) belonged to as yet unidentified families of *M. tuberculosis *or had individual non-clustering genotypes. Mutations conferring rifampicin and isoniazid resistance were detected in 49% and 54% isolates, respectively. Mutations in codon 531 of the *rpoB *gene and codon 315 of the *katG *gene were predominant among drug-resistant isolates. An association was found for belonging to the LAM strain family and having multiple drug resistance (R = 0.27, p = 0.0059) and also for the presence of a mutation in codon 531 of the *rpoB *gene and belonging to the Beijing strain family (R = 0.2, p = 0.04).

**Conclusions:**

Transmission of drug-resistant isolates seems to contribute to the spread of resistant TB in this oblast. The Beijing genotype and LAM genotype should be seen as a major cause of drug resistant TB in this region.

## Background

Tuberculosis remains to this day the most widespread infectious disease in many parts of the world and has high mortality rate. Among the estimated 9 million new cases that are annually recorded, antibiotic drug resistance of *Mycobacterium tuberculosis *has become an increasing problem. The appearance of multidrug-resistant (MDR)-TB and, more recently, of extensively drug-resistant (XDR)-TB is an actual threat to achieve TB control and elimination. Each year over 400,000 new cases of MDR-TB occur and, in every setting XDR cases are recognized (although their number is currently unknown) [1]. The use of molecular genetic methods to analyze the population structure of *Mycobacterium tuberculosis *has enabled data to be collected on the relationship between a specific genotype and its influence on the course of disease [2,3] with the outcome of disease being determined to a large extent by the individual characteristics of the patient and the genetic type of the mycobacterium [4]. Different genotypic families of *M. tuberculosis *have varying degrees of virulence eliciting different immune responses [5], which can be manifested as increased abilities of individual genetic types to acquire drug resistance. This can also affect the results of diagnostic tests, e.g., the interferon-gamma T-cell test [2,3].

Several researchers have shown a relationship between the genotype of isolate, drug resistance, and the type of genetic mutations responsible for the drug resistance. Isolates of *M. tuberculosis *belonging to the Beijing strain family are associated with drug resistance in Iran, Afghanistan, and Russia [6-8]. Thus, a detailed study of regional population structures of *M. tuberculosis *and identification of the specific genotypes associated with drug resistance can facilitate both a more effective drug therapy regime and give information at the molecular-epidemiological level.

A rapid determination of the resistance profile to anti-tuberculosis drugs defines the choice of an effective drug therapy regime. Molecular genetic methods for determining drug resistance of mycobacteria require minimum time for completion of an analysis and are especially effective when access to a bacteriological laboratory is limited. However, the use of these methods requires detailed information on the molecular mechanisms for developing drug resistance and the spectrum of mutations causing resistance. The *M. tuberculosis *genome is exceptionally conserved with minimal horizontal transfer and an absence of plasmids [9,10]. The acquisition of resistance is due to mutations and deletions within the chromosome. These chromosomal changes affect protein-binding structures for the drug or the activity of enzymes that metabolize the drug. Mutations and deletions in intergenic regions of the genes *katG*, *inhA*, and *oxyR-ahpC *are known to cause resistance to isoniazid. Resistance to rifampicin is most often caused by different mutations and deletions in the 81 bp catalyst region of the *rpoB *gene that encodes the β-subunit of DNA-dependent RNA-polymerase. These aforementioned mutations are the principal modes of drug resistance in *M. tuberculosis*.

A persistent increase of tuberculosis cases has recently been noted in the Ukraine. In 2004 TB incidence rate was 81 per 100,000 in a population and a mortality rate was 23 per 100,000 in a population. In 2009 TB incidence and a mortality rates were slightly decreased to 73/100000 and 18/100000, respectively [11]. The reported incidence of drug-resistant isolates of *M. tuberculosis *is growing steadily; however, data on the genetic variation of isolates of *M. tuberculosis *circulating in northern Ukraine and on the spectrum and frequency of occurrence of mutations determining resistance to the principal anti-tuberculosis drugs isoniazid and rifampicin have not yet been reported. Here we report for the first time, molecular typing of isolates circulating in Kharkiv Oblast using VNTR- and RFLP-typing methods. We also identified mutations in genes associated with the development of resistance to rifampicin and isoniazid, determined their frequency, and found correlations between having a specific mutation and belonging to a certain mycobacterial genotype.

## Methods

### Sampling

A total of 98 consecutive observed adult smear-positive pulmonary TB patients presenting to the State Clinical Anti-Tuberculosis Dispensary (SC ATD) No. 1 and its regional branches in Kharkiv, between January and March 2004 were enrolled in the study. The State Clinical Anti-Tuberculosis Dispensary is the main referral center for TB in Kharkiv and captures the majority of TB cases in the city. Males aged between 20 - 60 years (average age 40 years) dominated (81/98, 83%) the patient cohort. Smear - positive patients who had never been treated or were treated for less than 1 month were classified as new cases (n = 36, 37%), designed as first group of examined patients. The remaining patients (n = 62, 63%) were classified as chronic cases that previously had active TB and considered clinically cured but had become smear-positive again, designed as second group of examined patients. The information collected included socioeconomic and demographic characteristics, current and previous history of M. tuberculosis infection, history of contact with a TB case, history of previous anti-tuberculosis treatment and chest X- ray findings. The most common form was infiltrative tuberculosis of the lungs in 61/98 (62%) patients; fibrous-cavernous in 28/98 (29%) patients; disseminated tuberculosis of the lungs in 6/98 (6%) patients; caseous pneumonia in 2/98 (2%) patients; and exudative pleuritis in 1/98 (1%) patient. The study was approved by the Ethic Committee on Medical Research of the Institute of Chemical Biology and Fundamental Medicine SBRAS. Informed consent was obtained from all patients. There were no demographical or clinical differences between recruited patients and those who refused to participate. Bacteriological studies were performed at the Centralized Bacteriological Laboratory of SC ATD No.1, regularly fulfilling quality control. *M. tuberculosis *was grown for four weeks in Lowenstein-Jensen medium, before testing the isolates for sensitivity to isoniazid, rifampicin, streptomycin, ethambutol and kanamycin by the absolute concentration method [11].

### DNA isolation

DNA was isolated from *M. tuberculosis *cultures as described by van Soolingen [12].

### VNTR-typing

VNTR-typing of the loci; ETR A, B, C, MIRU2, MIRU4, MIRU10, MIRU16, MIRU20, MIRU23, MIRU24, MIRU26, MIRU27, MIRU31, MIRU39, and MIRU40 was performed by PCR with a final volume of 20 μL containing; 65 mM Tris-HCl (рН 8.9), 16 mM (NH_4_)_2_SO_4_; 2.5 mM MgCl_2_, 0.05% Tween 20, 0.2 mM dNTP, 1 μM of the appropriate oligonucleotide primers, 1 unit of Taq-DNA-polymerase, and 1-10 ng of *M. tuberculosis *genomic DNA. The oligonucleotide primer sequences are given in Table 1. The reaction was carried out in an iCycler amplifier (Bio-Rad, USA) where after an initial denaturation step of 96°C for 3 min, 33 cycles were performed as follows; denaturation at 95°C for 5 s, annealing at 60°C for 10 s, and elongation at 72°C for 20 s.

**Table 1:** Oligonucleotide sequences of primers used

Primer name	Nucleotide structure
ETRA1	5'-GATTGAGGGGATCGTGATTGG-3'
ETRA2	5'-CAGCTAGGCACTCCTGAGATTCC-3'

ETRB1	5'-BGCGAACACCAGGACAGCATCATG
ETRB2	5'-GGCATGCCGGTGATCGAGTGG

ETRC1	5'-CCTTATGCTTTGCCTGTTTGACC-3'
ETRC2	5'-TGTTCGGGGTGAGAAGATCG-3'

MIRU02U	5'-CAGGACACGGGTTCTACTG-3'
MIRU02R	5'-GGACTAGGTCGAGGTTGTGTC-3'

MIRU04U	5'-CAGGTCACAACGAGAGGAAGAGC
MIRU04R	5'-GCGGATCGGCCAGCGACTCCTC

MIRU10U	5'-GACTTCCAACAGCACCGTCTTATC-3'
MIRU10R	5'-TCGCACCGATCACGCTACG-3'

MIRU16U	5'-GTTGGAAACGGCGGTTATTGAC-3'
MIRU16R	5'-CGGAGTCGTCCAGCAAGACC-3'

MIRU20U	5'-TCGGAGAGATGCCCTTCGAGTTAG -3'
MIRU20R	5'-TCACGGTCTCCGCACTAACG-3'

MIRU23U	5'-CTCACCAGGATCGCCAAACC-3'
MIRU23R	5'-TCTGACTCATGGTGTCCAACC-3'

MIRU24U	5'-GCTTGTGCGGGAAGGCTA-3'
MIRU24R	5'-CGATCGCGGATCTTTGCT-3'

MIRU26U	5'-CCAGCAGTTGAGCACAGTTCG-3'
MIRU26R	5'-GGATAGGTCCGAGTTCGATTTCC-3'

MIRU27U	5'-CGGTGACCAACGTCAGATTC-3'
MIRU27R	5'-ACGTGACGGGGCATCTTC-3'

MIRU31U	5'-CCTTATGCTTTGCCTGTTTGACC-3'
MIRU31R	5'-TGTTCGGGGTGAGAAGATCG-3'

MIRU39U	5'-GTCAACAGACCACTAGACAAGCC-3'
MIRU39R	5'-GCAGCGTCCGTACTTCCG-3'

MIRU40U	5'-GCAAGAGCAAGAGCACCAAGC-3'
MIRU40R	5'-TGTCTAATCAGGTCTTTCTCTCACGC-3'

MSPA	5'-CGATCTGGTCGGCCCCGAAC-3'
MSPB	5'-TTCGTCGGGGTGTTCGTCCA-3'

MUT531U	5'-AAACCACAAGCGCCGAATGTC-3'
MUT531R	5'-TCTGATCGGCTCGCTGTC-3'

RPOB1	5'-AACCGCCGCCTGCGTACGGT-3'

The number of tandem repeat copies was calculated as a function of the size of the PCR product. The structure and number of repeat copies was also verified by direct sequencing of the amplified DNA fragments. The genotype of each isolate was expressed as a set of 15 digits where each digit of the 15-place number showed the number of copies of the corresponding tandem repeat.

### RFLP-typing

RFLP-typing of *M. tuberculosis *DNA isolates was performed using the method described by van Soolingen and van Embden [12-14].

### Statistical analysis

Statistical analysis was performed using the program Statistica 6.0 (Statsoft Inc.). Cluster analysis and construction of dendrograms was carried out using the UPGMA criterion (unweighted pair-group average) and the Neighbor-Joining method.

### Identification of mutations associated with the development of resistance to isoniazid and rifampicin

Primers were designed for amplification of fragments containing codon 315 of the *rpoB *gene (MspA and MspB), and codon 531 of the *rpoB *gene (MUT531U and MUT531R). The primer sequences are given in Table 1. PCR amplification was performed in 20 μL reaction volumes containing; 65 mM Tris-HCl (pH 8.9), 16 mM (NH_4_)_2_SO_4_, 0.05% Tween 20, 2.5 mM MgCl_2_, 100 μM dNTP, 1 μM primers, 1 unit of Taq-DNA-polymerase, and 1-10 ng of *M. tuberculosis *genomic DNA. The reaction was carried out in a Tertsik amplifier (DNA-technology, Russia) with an initial denaturation step of 96°C for 3 min, followed by 38 cycles consisting of: denaturation at 95°C for 10 s, annealing at 68°C for 10 s (for the fragment of *katG*), or at 55°C for 10 s (for the fragment of *rpoB*), and elongation at 72°C for 20 s. The presence of an amplification product was checked by electrophoresis in 6% PAAG with subsequent visualization of the DNA by ethidium bromide staining.

A mutation in codon 315 of the *katG *gene was determined by hydrolyzing 10 μL of the amplification product using the enzyme MspI. The total reaction volume was 15 μL. One unit of enzyme was added twice at 1-hour intervals and the mixture was incubated at 37^о^C. The restriction mixture was analyzed by electrophoresis in 8% PAAG. Fragments of 72 bp, 57 bp, and 6 bp corresponded to the wild-type allele of *katG*; whilst fragments of 72 bp, 36 bp, 21 bp, and 6 bp corresponded to mutations in codon 315 of *katG*.

The amplification product from *rpoB *was hydrolyzed by endonuclease restriction using the enzyme BstPAI in order to determine whether a mutation in codon 531 of gene was present. In case where a wild-type allele was present in the amplified DNA fragment, a substitution was inserted into one of the oligonucleotide primers to create a recognition site for the endonuclease. The hydrolysis was carried out in a 25 μL reaction volume using 20 μL of the amplification product and 2 units of BstPAI. The reactions were incubated at 65°C for 2 hours and the restriction products analyzed by electrophoresis in 8% PAAG with subsequent visualization of the DNA by ethidium bromide staining. Fragments of 153 bp corresponded to the mutant-type allele; fragments of 135 bp and 18 bp to a wild-type allele. Direct sequencing of the catalyst region of the *rpoB *gene was performed on an ABI 3130XL Genetic Analyzer automated sequencer (Applied Biosystems, USA) using the Big dye 3.1 set and primers RPOB1 and MUT531R (see Table 1).

## Results

### VNTR-typing

**Figure 1:**
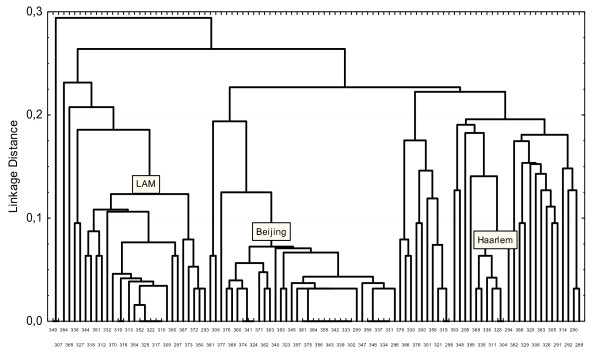
**Clustering dendrogram of 98 isolates of the *M. tuberculosis *complex isolated in Kharkov Region constructed from results of VNTR-typing**. The abscissa axis is number of isolate. The ordinate axis is a linkage distance.

VNTR-typing using 15 polymorphic loci of the 98 *M. tuberculosis *isolates identified 75 genetic types. A total of 67 isolates had unique profiles. The remaining 31 isolates formed clusters of different sizes (with a difference coefficient <0.125). The Hunter-Gaston discrimination index (HGDI) for this method was 0.985. A clustering dendrogram of *M. tuberculosis *isolates obtained from tuberculosis patients in Kharkiv Oblast (Figure 1) was constructed using the UPGMA and N-J methods based on the VNTR-typing and shows three large branches. These branches consist of smaller clusters or in most instances, include isolates with unique allele profiles.

The Beijing strain family is represented in this study by 32 isolates (32%). Within the Beijing strain family isolates, the majority 10 (31.5%) had the genetic profile 233325153533424 (for typing over 12 MIRU-loci and three ETRA, B, C-loci). The remaining isolates of belonging to this family had either a unique profile or fell into small clusters of 3-5 isolates.

Representatives of the LAM isolates family formed a large cluster of 23 isolates (23%). Of these, 5 isolates had the allelic profile 134325153225222, and 2 isolates 134325113225222. The others isolates of LAM families had unique profiles.

The Haarlem strain family was represented by 7 isolates (7%), two isolates had the same genetic profile, 235325153323323; but the allelic profiles of the other five isolates were unique.

We were unable to assign 36 isolates to any previously known families. A small cluster was formed by 4 (4%) of the isolates but the remaining 32 (32%) had a unique genetic profiles.

Isolates of the Beijing strain family were most common among the first group of examined patients (31%), followed by isolates of the LAM strain family (14%), and finally isolates of the Haarlem strain family (8%). In the second group isolates of the Beijing and LAM strain families represented 39% and 29% of all isolates, respectively. The increase in the number of isolates from the Beijing and LAM strain families was not statistically significant (χ^2 ^= 0.33, p = 0.56; χ^2 ^= 2.08, p = 0.15, respectively).

### RFLP-typing

**Figure 2:**
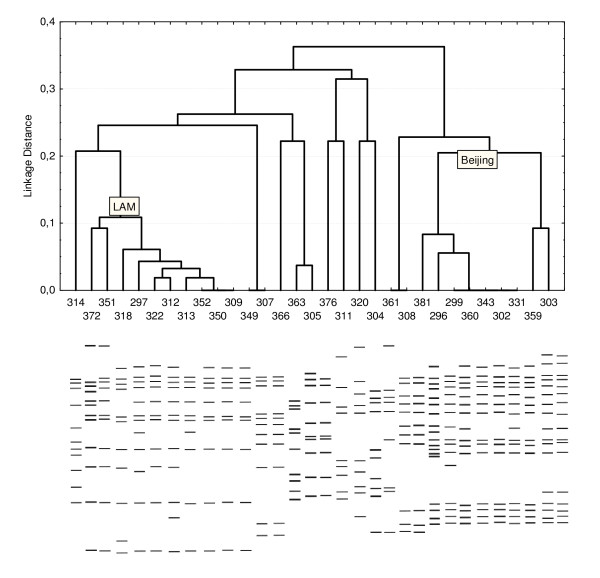
**Clustering dendrogram of 31 isolates from *M. tuberculosis *constructed from results of IS6110-RFLP-typing**. Tracks 381, 296, 299, 360, 343, 302, 331 are isolates belonging to the Beijing strain family; tracks 372, 351, 318, 297, 322, 312, 313, 352, 350, 309, are isolates belonging to the LAM strain family. The abscissa axis is number of isolate. The ordinate axis is a linkage distance. Simulation of RFLP-typing of these samples appears below.

A total of 31 isolates with sufficient DNA for analysis were typed using the RFLP-IS6110 method for a more accurate characterization. A schematic representation of the results is shown in Figure 2. The 31 isolates were represented by 23 different genetic profiles using RFLP-IS6110 typing versus 26 profiles obtained from VNTR-typing of the same samples. A clustering dendrogram for these isolates was constructed based on the results of the IS6110-RFLP-typing (Figure 2). Two large clusters with a mutual difference coefficient of <0.225 are visible on the dendrogram. The clustering was 29%.

The 9 isolates of the Beijing strain family made up a very large and rather homogeneous group (figure 2, lanes 381 to 303) and had between 14 and 19 copies of the IS6110 element. We identified several variants of the Beijing strain family; 2 isolates for variant B; 6 isolates for variant A, and 1 isolate for variant C [15]. All 9 isolates had a characteristic VNTR-profile of Beijing family. A heterogeneous group (Figure 2, lanes 372 to 309) with a difference coefficient <0.1, that included isolates of the LAM strain family and made up 30% (10 samples) was also seen. The presence of 10 to 12 copies of IS6110 was characteristic of this family. VNTR-typing confirmed that these isolates belong to the LAM family. Two isolates (Figure 2, lanes 307 and 349) had the same RFLP- and VNTR-profile and formed a cluster. Three isolates (Figure 2, lanes 320, 311 and 304) had same RFLP-profiles, but only two isolates (№№311,304) were assigned according to the Haarlem strain family based on VNTR-typing. Isolates № 361 and №308 had identical RFLP-profiles and possessed by VNTR types. The remaining 5 isolates (30%) were found to have the unique RFLP- and VNTR-profiles.

**Figure 3:**
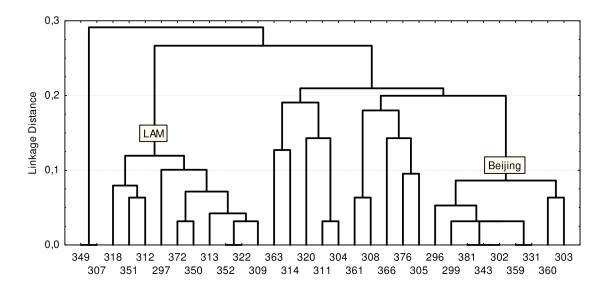
**Clustering dendrogram of 31 isolates from *M. tuberculosis *constructed from results of VNTR-typing**. The abscissa axis is number of isolate. The ordinate axis is a linkage distance.

A clustering dendrogram of these same isolates was also constructed based on VNTR-typing of these isolates (Figure 3). The difference coefficients for the LAM and Beijing strain families were <0.1 and <0.125 respectively. We computed the Hunter-Gaston discrimination indices for both methods: for RFLP-typing, it was 0.97; for VNTR-typing, 0.987. This indicated that VNTR-typing using 15 loci had a higher resolving capability.

Here the perfect concordance between two highly discriminatory molecular methods was observed. Rand coefficient commonly used for comparing congruence of typing methods was 0.963 [16].

### Analysis of drug resistance

All isolates were tested using the absolute concentration method for resistance to isoniazid, rifampicin, streptomycin, kanamycin and ethambutol. Monoresistant tuberculosis was observed in 14/98 (14%) patients and polyresistant, in 60/98 (61%). It was found that 58% (57/98) of the isolates were resistant to isoniazid, 49% (48/98) to rifampicin, 75% (74/98) to streptomycin, 40% (39/98) to kanamycin and 35% (34/98) to ethambutol. MDR-TB (resistance to isoniazid and rifampicin, both) was seen in 48/98 (49%) isolates [17]. Resistance to all tested drugs was seen in 30/98 (30%) isolates. Twenty four isolates (25%) were sensitive to all drugs tested.

### Determination of mutations associated with resistance to rifampicin and isoniazid

PCR-RFLP analysis showed that 35 isolates (73%) that were resistant to rifampicin had the codon substitution Ser531Leu in *rpoB*. Sequencing of an 81 bp region of *rpoB *was performed for 13 rifampicin-resistant isolates with no mutation present at codon 531 of the gene (Tables 2). Codon substitutions were identified in five locations; in 6 isolates (12.5%) codon 526 changed from His→Tyr, in 4 insolates (8%), codon 516 changed from Asp→Val, and in 2 isolates (4%), codon 533 changed from Leu→Pro. In 1 isolate (2%), two mutations were observed simultaneously in codons 509 and 511 with changes from Ser→Thr and Leu→Pro respectively. The sensitivity and specificity of the genetic analysis was 100%.

**Table 2:** Codon substations other than Ser531Leu found when sequencing the *rpoB *gene from isolates resistant to rifampicin

**Strain No**.	Codon, substitution
	509 AGC→ACC, Ser→Thr
337	511CTG→CCG, Leu→Pro

380	516 GAC→GTC, Asp→Val

372	516 GAC→GTC, Asp→Val

319	516 GAC→GTC, Asp→Val

367	516 GAC→GTC, Asp→Val

358	526 CAC-→ CTC, His→Leu

378	526 CAC→CTT, His→Leu

374	526 CAC→TAC, His→Tyr

322	526 CAC→TAC, His→Tyr

379	526 CAC→TAC, His→Tyr

290	526 CAC→TAC, His→Tyr

368	533 CTG→CCG, Leu→Pro

301	533 CTG→CCG,Leu→Pro

Screening of the *M. tuberculosis *isolates for the presence of a mutation in codon 315 of the *katG *gene showed that 53/57 (93%) isoniazid-resistant isolates carried this mutation. Four isoniazid- sensitive isolates also had the Ser315Thr substitution. Repeated analysis of these isolates using DNA sequencing of the corresponding part of the gene confirmed that a mutation was present in this codon. This may indicate that the results of the bacteriological study were flawed. The sensitivity and specificity of the genetic analysis for resistance to isoniazid was 93% and 90%, respectively. Our findings in this study agree with reports in the literature where the phenotype test for determining sensitivity to isoniazid has also been shown to be unreliable (92%) [18,19].

### Analysis of the correlation between multiple drug resistance and genotype of the isolates

The frequency and spectrum of drug resistance of the isolates belonging to the different strain families were analyzed (Table 3). Belonging to the LAM strain family and having multiple drug resistance was shown to be associated using the Spearman correlation coefficient (R = 0.27, p = 0.0059). Such an association was not found for the Beijing strain family (R = 0.1, p = 0.3241).

**Table 3:** Analysis of drug resistance of isolates belonging to different strain families of *M. tuberculosis*

	Total	Beijing	%	LAM	%	Haarlem	%	Others	%
**Total**	**98**	**31**	**32**	**16**	**16**	**7**	**7**	**44**	**45**

**Sens. to all AT drugs**	**22**	**6**	**27**	**4**	**18**	**1**	**5**	**11**	**50**

**Resistant to 1-4 AT drugs**	**76**	**25**	**33**	**12**	**16**	**6**	**8**	**33**	**43**

**MDR**	**48**	**17**	**35**	**11**	**23**	**1**	**2**	**19**	**40**

AT = anti-tuberculosis, MDR = multiple drug resistance

## Discussion

Mutations in the catalyst region of the *rpoB *gene are associated with the development of resistance to rifampicin. In 86% of occurrences, they arise at codons 531, 526, or 516. These mutations in certain regions of Russia are responsible for almost all rifampicin-resistant isolates [20]. In our collection of isolates, mutations at these codons were identified in 94% of the rifampicin-resistant isolates, which is similar to other reported results [14] and reflects the geographical similarity of these regions. The number of rifampicin-resistant isolates with mutations at codons 531, 526, and 516 of the *rpoB *gene (73%, 12.55% and 8% respectively) agreed with those found by Nikolaevskyy et al. during typing of isolates from Nikolaev and Odessa, Ukraine [21]. These results are typical of countries with an average or high level of resistance to rifampicin and predominance of isolates belonging to the Beijing strain family [22].

The occurrence of the nucleotide substitution AGC→ACC at codon 315 of the *katG *gene in isoniazid-resistant isolates correlated well with the phenotype test (93%). Similar results have been obtained for northwest Russia (93.6%), Latvia (91.0%), and Lithuania (85.7%) [22-24]. It is noteworthy that the aforementioned mutation according to the literature has an insignificant effect on decreasing bacterial adaptation and is very often encountered in rifampicin-resistant strains [23,25,26]. It is necessary to note, a considerable quantity isolates (30/98, 30%) were resistant to all tested drugs, however we were not able to designate these isolates as XDR due to lack of fluorquinolones resistance data [27,28].

Genotyping of 15 polymorphic loci showed a large variation in the genetic profiles of the studied isolates. The high discrimination level of VNTR-typing using 15 polymorphic loci (HGDI = 0.985) resulted in a significant percentage of isolates (36%) belonging to as yet unidentified strain families of *M. tuberculosis *or having individual non-clustering genotypes. Six loci (MIRU10, MIRU26, MIRU31, MIRU39, MIRU40 and ETRA) had a discrimination index >0.5. The number of allele repeats varied from 3 to 9, confirming their high discriminating capability [29]. The discovery of a large cluster of isolates belonging to the Beijing strain family with numerous branches on the clustering dendrogram indicates that these isolates are widely distributed and have circulated in this region for a long time. The frequency of occurrence of this strain family was 34%, which is below the frequency of occurrence of isolates of this strain family for the Russian Federation (about 50%) [30,31]. The frequency of occurrence of *M. tuberculosis *isolates from the LAM strain family (23%) in Kharkiv Oblast was comparable with that found for Bulgaria (22%) [32] and Kaliningrad Oblast of Russia (18%) [33]. However, isolates from this strain family in the Urals and western Siberian regions of Russia were only 9-10% [31,34]. We observed that just 7% of our isolates were from the Haarlem strain family, which has European origins. The frequency of occurrence of this family is higher in European countries and in an analogous study in Scandinavia the distribution of isolates from the Haarlem strain family was 20% [35]. The following migration patterns for the various strain families of *M. tuberculosis *can be proposed: strains from the Beijing family were distributed into Ukraine from eastern Russia; whilst strains from the LAM and Haarlem families, came from the European side.

We demonstrated an association between belonging to the LAM strain family and having multidrug resistance (MDR). At the same time such association had not been found for the Beijing strain family. Previously, not only the predominance of the Beijing strain family but also a statistically significant association for this family with MDR (89 of 225 isolates belonging to the Beijing family; of these 48.3% had MDR, 19.8% did not; RR 2.43, 95% CI 1.63-3.63) was described during a study of isolates from *M. tuberculosis *circulating in Odessa and Nikolaev (Ukraine) [21]. Moreover, the previous study showed that isolates with the genetic profile 223325173533424 and the Beijing strain family subtype had more mutations associated with rifampicin and isoniazid resistance than those from other subtypes of this family (RR 1.86, 95% CI 1.41-2.47 and RR 1.74, 95% CI 1.17-2.57, for isoniazid and rifampicin resistance, respectively). Drobniewski et al. showed during typing of *M. tuberculosis *circulating in Russia that this genotype was frequently encountered among prisoners [36]. In our study we didn't find isolates with such genetic profile (223325173533424). Isolates with the genetic profile (233325153533424) make up 31.5% of all isolates from the Beijing isolates family in our setting. However, reliable differences between other Beijing isolates family subtypes with respect to the predominance of mutations related to isoniazid and rifampicin resistance were not found (χ^2 ^= 1.035, p = 0.3; χ^2 ^= 0.68, p = 0.4, respectively).

We did not find a correlation between having a substitution at codon 315 of the *katG *gene and belonging to the Beijing or LAM strain family (R = 0.14, P = 0.17, R = 0.08, P = 0.44, respectively). However, a correlation was found between having a mutation at codon 531 of the *rpoB *gene and belonging to the Beijing strain family (R = -0.2, P = 0.04). This correlation was not found for the LAM strain family (R = 0.19, P = 0.06). It is interesting to note that according to the literature the presence of such associations depends on the geographic region because an association between belonging to the Beijing strain family and having a mutation at codon 531 of the *rpoB *gene was not found in eastern Asia [37]. This association is encountered in northwestern Russia but in northern Africa is more often found in isolates belonging to the Beijing strain family than in those of non-Beijing strain families [30,38]. An association was also found between having a substitution at codon 315 of the *katG *gene and belonging to the Beijing strain family in studies carried out in Russia and Kazakhstan [30,39].

A study of *M. tuberculosis *genotypes in patients with newly acquired tuberculosis and chronic tuberculosis is very important for the epidemiological analysis of genotype distribution in a specific region. We found a percentage increase of the Beijing and LAM isolates in the second group of patients at comparison with the first ones. However, it was not statistically significant (χ^2 ^= 0.33, p = 0.56; χ^2 ^= 2.08, p = 0.15, respectively). This may indicate that these strain families of *M. tuberculosis *are transmitted to an equal extent.

## Conclusions

Here we have characterized for the first time, the genetic variation of *M. tuberculosis *isolates circulating in northeastern Ukraine and determined the frequency of occurrence of mutations associated with the development of resistance to isoniazid and rifampicin. Associations between MDR of the isolate and belonging to the LAM strain family and between belonging to the Beijing strain family and having a mutation at codon 531 of the *rpoB *gene were demonstrated. Representatives of the LAM strain family were genetically heterogeneous in the studied isolates, which is inconsistent with recent transmission and has high epidemiological significance for this region. Unfortunately, this particular study was rather small and the high percent of secondary cases could bias real population structure of Mycobacterium tuberculosis complex strains from Ukraine. We suggest that further DNA fingerprinting studies involving better characterized prime and secondary TB cases obtained more recently will be useful. The discovery of the principal phylogenetic families of *M. tuberculosis *and the description of their properties, especially drug resistance, will help the selection of more effective preventative measures in the fight against tuberculosis in the complicated epidemic situation in Ukraine.

## Competing interests

The authors declare that they have no competing interests.

## Authors' contributions

MAD carried out the molecular genetic studies, drafted the manuscript, and performed the statistical analysis. AAL, PIP, VSK participated in acquisition of sample collection. EAK participated in the sequence alignment. MLF conceived of the study, and participated in its design and coordination. All authors read and approved the final manuscript.

## Pre-publication history

The pre-publication history for this paper can be accessed here:

http://www.biomedcentral.com/1471-2334/11/77/prepub
